# Endoscopic mucosal resection – transoral outlet reduction: treatment of excessively wide gastro-jejunal anastomosis

**DOI:** 10.1055/a-2587-8447

**Published:** 2025-05-06

**Authors:** Mathilde Sanavio, Sonia Carparelli, Jean-Michel Gonzalez, Marc Barthet

**Affiliations:** 1 Gastroenterology, Hepatology and Transplantation Department, Hopital Saint Eloi, Montpellier, France; 2 9344 Division of Gastroenterology and Digestive Endoscopy, fondazione I.R.C.C.S, San Giovanni Rotondo, Italy; 3 37059 Department of Hepatogastroenterology, Assistance Publique des Hôpitaux de Marseille, Aix-Marseille Université, Hôpital Nord, Marseille, France

Excessively wide gastro-jejunal anastomosis (GJA) after gastric bypass can result in weight gain or refractory dumping syndrome. We propose an alternative endoscopic procedure to the so-called TORe (Transoral Outlet Reduction) performed by endoscopic suturing of the GJA.

The first case involved a 36-year-old woman with grade III obesity who had undergone multiple bariatric surgeries (gastric band placement and gastric bypass). Initially, significant weight loss has been obtained. However, 8 years later, she lost all her weight. Additional examinations were conducted to assess the surgical configuration. Gastroscopy revealed a nonstraight anastomosis.

The second case involved a 68-year-old man who had undergone repeated operations for a hiatal hernia with significant GERD, which led to a GJA. Since this surgery, he no longer has GERD, but a severe dumping syndrome is present at every meal 9 years later. Gastroscopy revealed a huge enlargement of GJA.

Therefore, we performed both the new endoscopic procedures that we call EMR-TORe.

This procedure regained almost involved a circular mucosectomy at the anastomosis site, performed using a large-channel operative endoscope, under CO _2_ insufflation, with a Duette (Wilson cook) elastic band ligature system ( Video 1 ). The diet was initially liquid and then blended, and a proton pump inhibitor treatment was initiated for 2 months. The patient was followed up every month clinically and a new endoscopy was realized at 2 months. The first patient has lost 8 kg and the second no longer has dumping syndrome.

This is the EMR-TORe endoscopic procedure. Using a large-channel operative endoscope, with a duette elastic band ligature system, after injection of adrenaline serum, a circumferential mucosectomy of the gastrojejunal anastomosis is performed from proximal to proximal.Video 1

This technique has been developed for the treatment of GERD 1 with an incomplete circumferential EMR to avoid stenosis, called in this indication ARMS. Nevertheless, the stenosis rate is approximately 20%. In these patients, a complete EMR was performed to narrow as much as possible the GJA, inducing a fibrotic scar. Thus, endoscopically the two anastomoses have been narrowed leading to an accurate clinical result.

Endoscopy_UCTN_Code_TTT_1AO_2AN

## Notes

Endoscopy E-Videos https://eref.thieme.de/e-videos
*E-Videos* is an open access online section of the journal *Endoscopy* , reporting on interesting cases and new techniques in gastroenterological endoscopy. All papers include a high-quality video and are published with a Creative Commons CC-BY license. Endoscopy E-Videos qualify for HINARI discounts and waivers and eligibility is automatically checked during the submission process. We grant 100% waivers to articles whose corresponding authors are based in Group A countries and 50% waivers to those who are based in Group B countries as classified by Research4Life (see: https://www.research4life.org/access/eligibility/ ). This section has its own submission website at https://mc.manuscriptcentral.com/e-videos .
